# 

**Published:** 1890-03

**Authors:** 


CONTENTS.

Vol. 37 No. 3.

| | PAGE. |
|---|---|
| Recasting the Creed | 49 |
| The Drinking Habit | 50 |
| To a Hopeless Dyspeptic | 51 |
| Farmers and Farming | 54 |
| Apoplexy | 56 |
| Troubled Topolobampo | 57 |
| Wheat Meal vs. White Flour | 59 |
| A mistake to Exercise for Strength alone | 60 |
| Would you like a New Nose? | 61 |
| A Strange Coincidence | 62 |
| Insomnia | 63 |
| The office of Iron in the Blood | 63 |
| Source of Colors | 64 |
| Old and Young Sleeping Together | 64 |
| Where some Nuts Come From | 65 |
| Confectioners' Colors | 66 |
| The Ear | 67 |
| Effect of Beer Drinking | 67 |
| Pure Air | 67 |
| Bacilli on a Bald Head | 68 |
| In the Sick Room | 68 |
| How to Elude the Doctor | 69 |
| Household | 70 |
| Miscellaneous | 71 |
| Literary | 72 |

INDEX TO ADVERTISEMENTS OF HALF A PAGE AND OVER.

| | PAGE. |
|---|---|
| An Unprecedented Offer | I |
| Celluloid | II |
| Hollow Suppositories | III |
| Christ Before Pilate | IV |
| Lactopeptine | V |
| E. C. Morris & Co | VI |
| Dennin's Certain Cure | VII |
| Revised Premium List | VIII |
| The Herb Cure Co | X |

